# Protective Behaviors Against COVID-19 by Individual Vaccination Status in 12 Countries During the Pandemic

**DOI:** 10.1001/jamanetworkopen.2021.31137

**Published:** 2021-10-26

**Authors:** Rafael Goldszmidt, Anna Petherick, Eduardo B. Andrade, Thomas Hale, Rodrigo Furst, Toby Phillips, Sarah Jones

**Affiliations:** 1 Brazilian School of Public and Business Administration, Getulio Vargas Foundation, Rio de Janeiro, Brazil; 2 Blavatnik School of Government, Oxford University, Oxford, United Kingdom; 3 Imperial College, London, United Kingdom

## Abstract

This cross-sectional study examines associations of protective behaviors against COVID-19 among individuals in 12 countries who had received 0, 1, or 2 COVID-19 vaccine doses.

## Introduction

The eagerly awaited onset of vaccination programs against COVID-19, along with people’s urge to return to “normal life,” has prompted concerns that individuals who were vaccinated would reduce their protective behaviors faster than recommended.^1,2^ Whereas worries about risk compensation^3^ have proven unfounded in some preventive medicine contexts (eg, human papillomavirus vaccination^4^), signs of riskier conduct among the treated have appeared in others (eg, individuals who use HIV preexposure prophylaxis^5^). Based on representative samples of 12 countries in various stages of immunization programs, this study compared the self-reported protective behaviors of individuals who had received 0, 1, or 2 COVID-19 vaccine doses. We assessed the period February 23 to June 1, 2021, when, barring anecdotal exceptions,^6^ governments had yet to exempt individuals who had been vaccinated from COVID-19 protective-behavior policies.

## Methods

This cross-sectional study was approved by the ethical review boards of Imperial College London (ICL) and Columbia University. Data on COVID-19 protective behaviors, vaccination status, and demographic characteristics were obtained from the ICL-YouGov Global Survey. Respondents completed written informed consent documents online. This study is reported following the Strengthening the Reporting of Observational Studies in Epidemiology (STROBE) reporting guideline.

Nationally representative samples were collected online in 15 countries, in biweekly waves. Countries with less than 5% of the population having received 2 doses of a COVID-19 vaccine by June 1 (Australia, Japan, and South Korea) were excluded, leaving 80 305 respondents from Canada, Denmark, France, Germany, Israel, Italy, Norway, Singapore, Spain, Sweden, the United Kingdom and the US (Table).

**Table.** Characteristics of Vaccinated and Unvaccinated Respondents

Characteristics	COVID-19 vaccine status, No. (%)		
	0 Doses (n = 54 846)	1 Dose (n = 14 989)	2 Doses (n = 10 470)
Age, y			
Mean (SD)	45.45 (15.85)	53.45 (17.16)	51.89 (18.41)
<30	10 975 (20.01)	1941 (12.95)	1649 (15.75)
30-45	15 511 (28.28)	2546 (16.99)	2127 (20.32)
45-60	16 099 (29.35)	3642 (24.30)	2479 (23.68)
≥60	12 261 (22.36)	6860 (45.77)	4215 (40.26)
Sex			
Women	28 910 (52.71)	7416 (49.48)	5276 (50.39)
Men	25 936 (47.29)	7573 (50.52)	5194 (49.61)
Chronic diseases, No.			
0	31 967 (58.29)	5788 (38.61)	4666 (44.57)
1	8609 (15.70)	3238 (21.60)	2007 (19.17)
≥2	6282 (11.45)	3294 (21.98)	2207 (21.08)
Missing	7988 (14.56)	2669 (17.81)	1590 (15.19)
People in household, No.			
1	10 835 (19.76)	3208 (21.40)	1600 (15.28)
≥2	42 865 (78.16)	11 618 (77.51)	8685 (82.95)
Missing	1146 (2.09)	163 (1.09)	185 (1.77)
Children in household, No.			
0	32 310 (58.91)	9608 (64.10)	5413 (51.70)
≥1	22 106 (40.31)	5296 (35.33)	4961 (47.38)
Missing	430 (0.78)	85 (0.57)	96 (0.92)
Mental health condition			
No	43 242 (78.84)	11207 (74.77)	8466 (80.86)
Yes	3616 (6.59)	1113 (7.43)	414 (3.95)
Missing	7988 (14.56)	2669 (17.81)	1590 (15.19)
Employment status			
Full-time	24 161 (44.05)	5340 (35.63)	4555 (43.51)
Part-time	6482 (11.82)	1829 (12.20)	1229 (11.74)
Full-time student	4573 (8.34)	551 (3.68)	430 (4.11)
Retired	8749 (15.95)	5246 (35.00)	3031 (28.95)
Not working	4268 (7.78)	964 (6.43)	536 (5.12)
Unemployed	5339 (9.73)	778 (5.19)	432 (4.13)
Other	1274 (2.32)	281 (1.87)	257 (2.45)
Health care worker			
No	29 275 (53.38)	6099 (40.69)	4332 (41.38)
Yes	1934 (3.53)	1188 (7.93)	1503 (14.36)
NA	23 637 (43.10)	7702 (51.38)	4635 (44.27)
Country			
Canada	4519 (8.24)	1945 (12.98)	184 (1.76)
Denmark	5281 (9.63)	920 (6.14)	810 (7.74)
France	5497 (10.02)	1075 (7.17)	475 (4.54)
Germany	5601 (10.21)	1064 (7.10)	343 (3.28)
Israel	472 (0.86)	285 (1.90)	2723 (26.01)
Italy	5731 (10.45)	871 (5.81)	414 (3.95)
Norway	5442 (9.92)	1214 (8.10)	353 (3.37)
Singapore	4872 (8.88)	846 (5.64)	1275 (12.18)
Spain	5821 (10.61)	880 (5.87)	344 (3.29)
Sweden	5300 (9.66)	1268 (8.46)	460 (4.39)
United Kingdom	2768 (5.05)	3073 (20.50)	1161 (11.09)
United States	3542 (6.46)	1548 (10.33)	1928 (18.41)
Trust in vaccines, mean (SD)^a^	2.72 (0.98)	3.22 (0.76)	3.39 (0.71)
Physical distancing, mean (SD)^a^	3.73 (1.03)	3.88 (0.90)	3.41 (1.15)
Wearing a mask, mean (SD)^a^	4.33 (1.25)	4.38 (1.14)	4.51 (1.05)

Abbreviation: NA, not available.

^a^ Scored on a 5-point scale, with higher score indicating more frequent behavior.

The ICL-YouGov Global Survey consists of repeated cross sections rather than longitudinal data, so weights from coarsened and exact matching were used to balance respondents who had not received a vaccine dose to those who had received 1 dose and respondents who had received 1 vaccine dose to those who had received 2 doses. Matching variables included calendar-week, age, sex, household size, employment status, employment in health care, trust in COVID-19 vaccines, having self-reported chronic diseases, having self-reported mental health conditions, and children in the household. We matched by country to generate a larger matched sample and by subnational region to provide more precise matching, although this consequently led to a smaller sample. The associations of vaccination status with COVID-19 protective behaviors, including physical distancing and mask wearing, were estimated using bivariate linear regressions with inverse probability of treatment weighting (eMethods in the Supplement). Protective behaviors were self-reported on a 5-point scale, with higher scores indicating more frequent behavior. Separate regression models were estimated for each dependent variable, and for each independent variable (binary indicators of first and second vaccine dose). Analyses were conducted using Stata statistical software version 14.1 (StataCorp). *P* values were 2-sided, and statistical significance was set at *P* = .05. Data were analyzed from May 10 to June 6, 2021.

## Results

Of 80 305 respondents included, 41 602 (51.8%) were women and 38 703 (48.2%) were men, and the mean (SD) age was 47.79 (16.81) years. Regression model estimates using the aggregated sample showed no difference in physical distancing among respondents who had received 1 vaccine dose compared with respondents who had not received a vaccine for country match (β = −0.02, 95% CI, −0.08 to 0.04]) or region match (β = −0.02; 95% CI, −0.08 to 0.04). There was a significant difference comparing individuals who had received 2 doses with those who had received 1 dose for country match (β = −0.08; 95% CI, −0.16 to −0.01) but not region match (β = −0.03; 95% CI, −0.13 to 0.06) (Figure). Respondents who had received 2 doses physically distanced less than respondents who had not received a vaccine according to country match (β = −0.07; 95% CI, −0.13 to <−0.01) and region match (β = −0.12; 95% CI, −0.21 to −0.02]) in an aggregated sample, matching respondents who had received 2 doses to those who had received none. In the US, there were similar differences in physical distancing among respondents who had received 2 vaccine doses vs those who had received 1 dose before (β = −0.22; 95% CI, −0.38 to −0.06]) and after (β = −0.33; 95% CI, −0.63 to −0.02]) the May 13 announcement of a vaccination-conditional protective-behavior policy (country match). There were no significant differences across groups for mask use using the aggregated sample.

**Figure.**
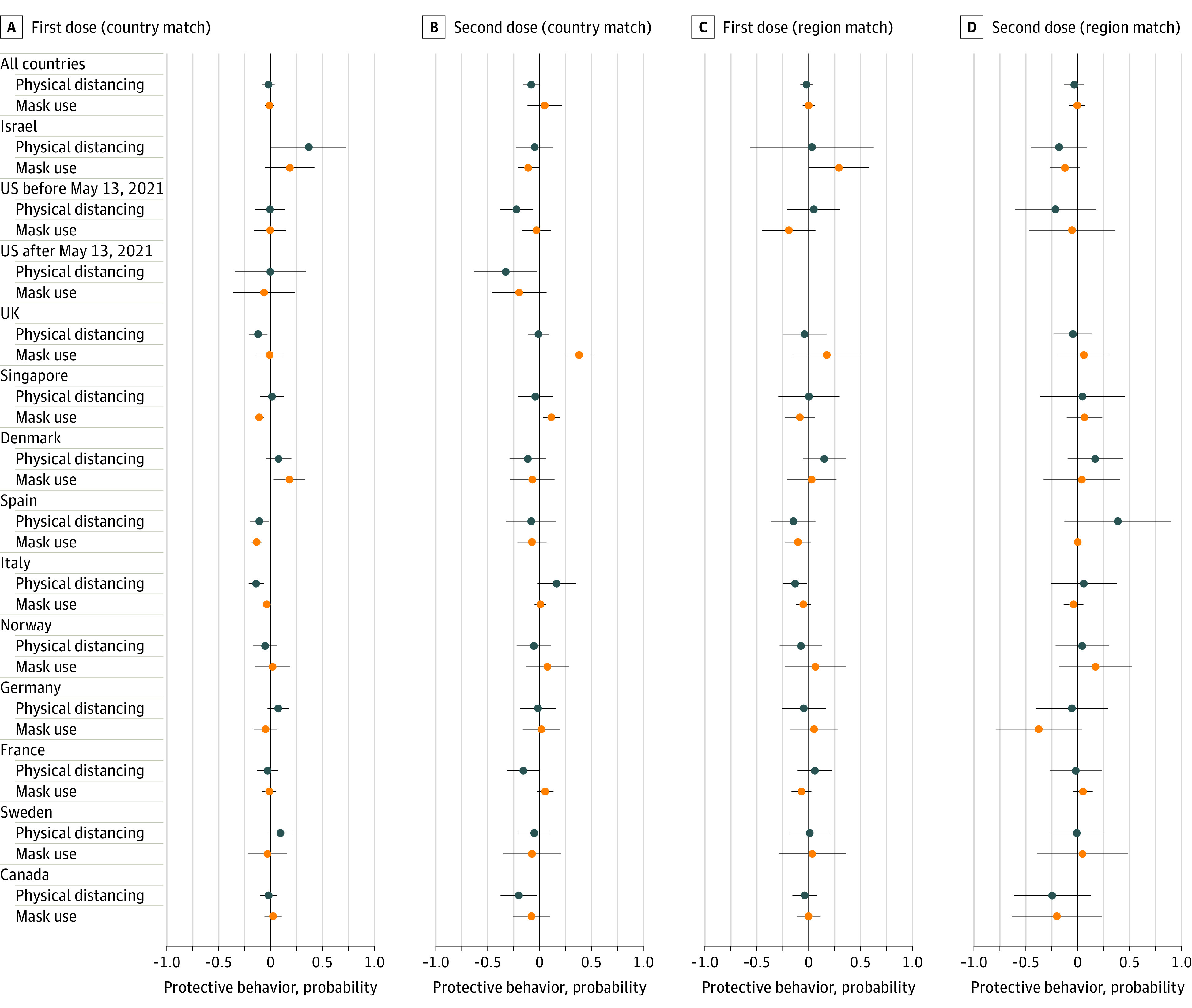
Estimates of Associations Between Vaccination and COVID-19 Protective Behaviors Countries are ordered by the proportion of the population who had received 2 vaccine doses as of June 1, 2021. The US sample is divided into periods before and after May 13, when individuals who were fully vaccinated were allowed to resume activities without wearing masks or physically distance. A and C, The aggregate-sample analysis includes 13 449 observations for country match (5229 individuals with 1 dose, 8220 individuals with 0 doses, and 56 386 unmatched observations) and 3698 for region match (1683 individuals with 1 dose, 2015 individuals with 0 doses, and 66 137 unmatched observations). For individual country matches, matched sample sizes ranged from 187 respondents (Israel) to 1614 respondents (UK). For subnational region matches, the matched sample size was smaller in Israel (66 respondents), while all other countries had matched samples of between 160 and 579 respondents. Estimates based on fewer than 20 matched observations are not reported, such as the US after May 13, 2021 for region match (13 observations). Models with the aggregated sample of all countries have clustered SEs at the country level. B and D, The analysis includes 6142 observations for country match (2893 individuals with 2 doses, 3249 individuals with 1 dose, and 19 317 unmatched observations) and 1842 observations for region match (915 individuals with 2 doses, 927 with 1 dose, and 23 617 unmatched observations). For country match, matched sample sizes ranged from 147 (US after May 13, 2021) to 1274 (UK) respondents. For subnational region match, the sample size was smaller in Spain (38 respondents), Germany (52 respondents), Canada (59 respondents), and Singapore (77 respondents), and other countries had samples of between 102 and 334 respondents. Results for the US after May 13, 2021 are not reported for region match because the sample size was fewer than 20 respondents (19 observations). Models with the aggregated sample of all countries have clustered SEs at the country level.

## Discussion

Despite occasional significant results, all of small magnitude, overall, this cross-sectional study found no substantial reduction in physical distancing or mask use associated with receipt of COVID-19 vaccine doses. This suggests that until early June, people generally did not engage in concerning levels of risk compensation as they acquired immunity. There was no discontinuity in behavior soon after US President Joe Biden announced that US adults who had been fully vaccinated need not wear masks or physically distance. We encourage investigation of the between-country variation identified in this study and reexamination of these results as time passes and as protective-behavior policies remain in place or alter in specificity and strength. Study limitations include the lack of longitudinal data and reliance on self-reported behavior.

## Notes

Supplement.
eMethods.
