# Supplementary material for: Protective Behaviors Against COVID-19 by Individual Vaccination Status in 12 Countries During the Pandemic
Source: JAMA Netw Open. 2021 Oct 26;4(10):e2131137. doi: 10.1001/jamanetworkopen.2021.31137 (PMC8548995; doi:10.1001/jamanetworkopen.2021.31137)
Supplement: Supplement. — eMethods. [file jamanetwopen-e2131137-s001.pdf]

Supplemental Online Content

Goldszmidt R, Petherick A, Andrade EB, et al. Protective behaviors against COVID-19 by individual vaccination status in 12 countries during the pandemic. *JAMA Netw Open*. 2021;4(10):e2131137. doi:10.1001/jamanetworkopen.2021.31137

eMethods.

This supplemental material has been provided by the authors to give readers additional information about their work.

© 2021 Goldszmidt R et al. *JAMA Network Open.*

eMethods.

Dependent Variables

Survey questions about physical distancing and mask wearing were introduced as follows: "Thinking about the last 7 days, how often have you taken the following measures to protect yourself or others from coronavirus (COVID-19)? As a reminder, please exclude any measures that you have already taken for reasons other than coronavirus (COVID-19)".

For physical distancing, the dependent variable was created as the average of responses to 5 questions that follow the above introductory sentence: i) avoided going out in general; ii) avoided going to shops; iii) avoided having guests to your home; iv) avoided small social gatherings (not more than two people); v) avoided medium-sized social gatherings (between 3 and 10 people). All 5 questions were scored on 5-point scales (1 - not at all, 2- rarely, 3 - sometimes, 4 - frequently and 5 - always). We did not include questions relating to large social gatherings and participation in public events in order to avoid the potential effects of vaccine passports that had, by the time of our study period, been issued for such gatherings in anecdotal cases (e.g. Israel, for theaters and concert halls). Our results hold, however, when these questions are included as well.

For mask use, the dependent variable was based on a single survey item, with the same aforementioned introduction: i) worn a face mask outside your home.

Vaccine Types

While we do not observe the specific vaccine taken by the respondents, by June 1, 22 single-shot vaccines (i.e. Johnson and Johnson) represented less than 4% of doses in the 23 US and less than 3% in the 6 other countries for which vaccine details are available.

© 2021 Goldszmidt R et al. *JAMA Network Open.*
